# Successful Small Intestine Colonization of Adult Mice by *Vibrio cholerae* Requires Ketamine Anesthesia and Accessory Toxins

**DOI:** 10.1371/journal.pone.0007352

**Published:** 2009-10-08

**Authors:** Verena Olivier, Jessica Queen, Karla J. F. Satchell

**Affiliations:** Department of Microbiology-Immunology, Feinberg School of Medicine, Northwestern University, Chicago, Illinois, United States of America; Duke University Medical Center, United States of America

## Abstract

*Vibrio cholerae* colonizes the small intestine of adult C57BL/6 mice. In this study, the physical and genetic parameters that facilitate this colonization were investigated. Successful colonization was found to depend upon anesthesia with ketamine-xylazine and neutralization of stomach acid with sodium bicarbonate, but not streptomycin treatment. A variety of common mouse strains were colonized by O1, O139, and non-O1/non-O139 strains. All combinations of mutants in the genes for hemolysin, the multifunctional, autoprocessing RTX toxin (MARTX), and hemagglutinin/protease were assessed, and it was found that hemolysin and MARTX are each sufficient for colonization after a low dose infection. Overall, this study suggests that, after intragastric inoculation, *V. cholerae* encounters barriers to infection including an acidic environment and an immediate immune response that is circumvented by sodium bicarbonate and the anti-inflammatory effects of ketamine-xylazine. After initial adherence in the small intestine, the bacteria are subjected to additional clearance mechanisms that are evaded by the independent toxic action of hemolysin or MARTX. Once colonization is established, it is suggested that, in humans, these now persisting bacteria initiate synthesis of the major virulence factors to cause cholera disease. This adult mouse model of intestinal *V. cholerae* infection, now well-characterized and fully optimized, should serve as a valuable tool for studies of pathogenesis and testing vaccine efficacy.

## Introduction

Innate immune defense against intestinal pathogens includes physical barriers, circulating factors, release of reactive oxygen species (ROS), and phagocytic cells. Evasion of these defenses by bacteria is essential to establish colonization followed by growth and expansion of the bacteria. *Vibrio cholerae* is a successful pathogen of the small intestine leading to a profuse watery diarrhea due to the action of cholera toxin (CT). The infectious dose for *V. cholerae* is relatively high due to poor acid tolerance in the stomach, the major physical barrier to *V. cholerae* infection [1]. Protective mechanisms by the bacteria against innate immunity in the gut include lipopolysaccharide (LPS), which provides resistance to complement [2], down-regulation of mannose-sensitive hemagglutinin leading to protection from non-specific IgA [3], formation of microcolonies within the crypts to protect from phagocytosis [4], and the action of secreted cytotoxins [5].

Although much is known about pathogenesis, detailed studies of the interaction of *V. cholerae* with the gut immune response resulting in clearance and/or protection have been limited by the lack of an animal model amenable to long-term colonization. Recently, we observed that C57BL/6 mice are susceptible to *V. cholerae* infection resulting in rapid lethality in a dose-dependent manner [6]. This rapid death occurred independently of CT and was due to the action of hemolysin (also known as *V. cholerae* cytotoxin) with a secondary contribution by the multifunctional, autoprocessing RTX toxin (MARTX) [6]. After low dose infection, mice survived and *V. cholerae* routinely colonized the small intestine and persisted for at least 72 hr. This ability to establish prolonged colonization depended on secreted accessory toxins as a multitoxin mutant strain deficient in production of CT and accessory toxins hemolysin, MARTX, and hemagglutinin (HA)/protease was cleared by 48 hr post-inoculation (p.i.) [5]. Although the accessory toxins play an important role in long-term colonization, a multitoxin mutant strain was immunogenic such that mice immunized with this strain were protected from subsequent lethal challenge with wild-type *V. cholerae* [6]. These studies established adult mice as a model system for study of *V. cholerae* host-pathogen interactions and vaccine efficacy and demonstrated a role for accessory toxins in cholera pathogenesis and disease dissemination.

As this mouse model has many potential applications, we revisited the development of the inoculation protocol to address which physical and genetic parameters most influence successful colonization of the mouse small intestine by *V. cholerae*. We find that successful colonization depends on anesthesia with ketamine-xylazine and neutralization of stomach acid with sodium bicarbonate, but not streptomycin treatment. Furthermore, we find that hemolysin and MARTX are each sufficient to promote prolonged colonization.

## Materials and Methods

### Bacterial strains and growth media

Bacterial strains used in this study are listed in Table 1. Internal deletions in genes *hlyA*, *hapA*, *rtxA*, *and lacZ* were created as previously described [7], [8]. *V. cholerae* is routinely grown on L agar plates with 100 µg/ml streptomycin and 50 µg/ml kanamycin as appropriate. Bacterial cultures for mouse inoculations were grown with shaking in L broth with antibiotics at 30°C to mid-log phase (A_600_≈0.5) or overnight (A_600_>6), washed with phosphate-buffered saline (PBS), and adjusted to desired CFU/ml based on the A_600_ determined using a Beckman Coulter DU530 spectrophotometer to estimate culture density.

**Table 1:** Bacterial strains used in this study.

Category	Strain Designation	Relevant genotype^a^	Reference
Wild-type *V. cholerae*	P27459	El Tor O1, Sm^R^	[23]
	E7946	El Tor O1, Sm^R^	[23]
	N16961	El Tor O1, Sm^R^	[8]
	O395	Classical O1, Sm^R^	[23]
	PL-21	O6	[24]
	1322-69	O37	[25]
	MO10	O139, Sm^R^	[26]
	203-93	O141	[27]
P4 mutant series	P4	P27459Δ*ctxAB*::Km^R^, Sm^R^	[28]
	KFV103	P4Δ*hlyA*	[7]
	KFV105	P4Δ*rtxA*	[7]
	KFV70	P4Δ*hapA*	[7]
	VOV3	P4Δ*hlyA*Δ*rtxA*	[6]
	KFV102	P4Δ*hlyA*Δ*hapA*	[6]
	KFV98	P4Δ*rtxA*Δ*hapA*	[6]
	KFV101	P4Δ*hlyA*Δ*rtxA*Δ*hapA*	[7]

a Sm^R^ = streptomycin resistant, Km^R^ = kanamycin resistant.

### Mouse intestinal inoculations

Mouse inoculations were performed according to Northwestern University IACUC approved protocols as previously described [5]. Briefly, specific pathogen free 4–5 week old C57BL/6, BALB/c, C3H (Harlan, Indianapolis, IN) or CD-1 (Charles River Laboratories, Wilmington, MA) were purchased and held in barrier housing until inoculated. Mice were given 0.1% (w/v) streptomycin for 4–7 days to ablate normal flora. 20–24 h prior to inoculation, food was removed from cages to empty the stomach. Mice were injected intraperitoneally with 60–70 mg/kg ketamine and 12–14 mg/kg xylazine. When mice were deeply sedated, 50 µl of 8.5% (w/v) NaHCO_3_ was administered orally immediately followed by 50 µl of bacterial suspension. After inoculation, mice were kept in microisolator cages with free access to food and sterile water without streptomycin. The optimized ABL inoculation protocol was performed as above except mice were not given streptomycin or food restricted prior to anesthetization and inoculation.

### Assessment of bacterial colonization

At designated time points after inoculation, mice were euthanized by cervical dislocation under anesthesia. The intestine above the caecum was dissected, homogenized in PBS, and then serially diluted in PBS for plate counts of recovered colony-forming units (CFU). Mice for which fewer than 10 colonies were recovered from 100 µl of undiluted homogenate (500 total CFU in the small intestine) were recorded as non-colonized and plotted below the detection limit line. Recovery of bacteria is reported as a Colonization Index calculated as Col. Index = CFU_recovered_/CFU_inoculated_.

### Statistical analysis

For statistical analysis, the Col. Index for non-colonizers was determined using actual recovered CFU (499 or less) or 1 if 0 colonies were recovered. All data were analyzed by one of two methods. A Kruskal-Wallis One Way ANOVA was performed to determine if any groups in a data set were significantly different followed by a Dunn's Multiple Comparisons post-test to identify significantly different groups. Identified groups were re-analyzed using a one-tailed Mann Whitney non-parametric t-test to compare medians. To determine if the frequency of survival or colonization in a group was significant, a chi-square (χ2) test was performed comparing colonized vs cleared or surviving vs severely moribund (euthanized) animals in the two groups. P values≤0.05 were considered significant. Results of the Mann Whitney or χ2 test are shown on figures as indicated in legend. All analyses were performed using Prism 4 for Macintosh (Graphpad Software, Inc., La Jolla, CA).

## Results

### Treatment of mice with ketamine-xylazine and sodium bicarbonate at time of inoculation are both necessary and sufficient for efficient intestinal colonization of mice

Previous studies showed that mice are susceptible to small intestine colonization with *V. cholerae* [5]. To better characterize oral infection of adult mice, we initiated these studies by determining which inoculation parameters facilitate successful colonization.

As a reference protocol based on previous experiments [5], [6], mouse inoculations were performed using streptomycin treatment (S), food restriction (F), anesthesia with ketamine-xylazine (A), neutralization of stomach acids with sodium bicarbonate (B), and inoculation of *V. cholerae* grown to mid-log phase (L). To determine to what extent each individual parameter contributes to colonization, mice were separated into 6 groups and 5 groups were inoculated with a high dose (1−5×10^7^ CFU) of El Tor O1 strain P27459 using 4 of the 5 parameters, while one control group was inoculated utilizing all 5 inoculation parameters (+all).

**Figure 1:**
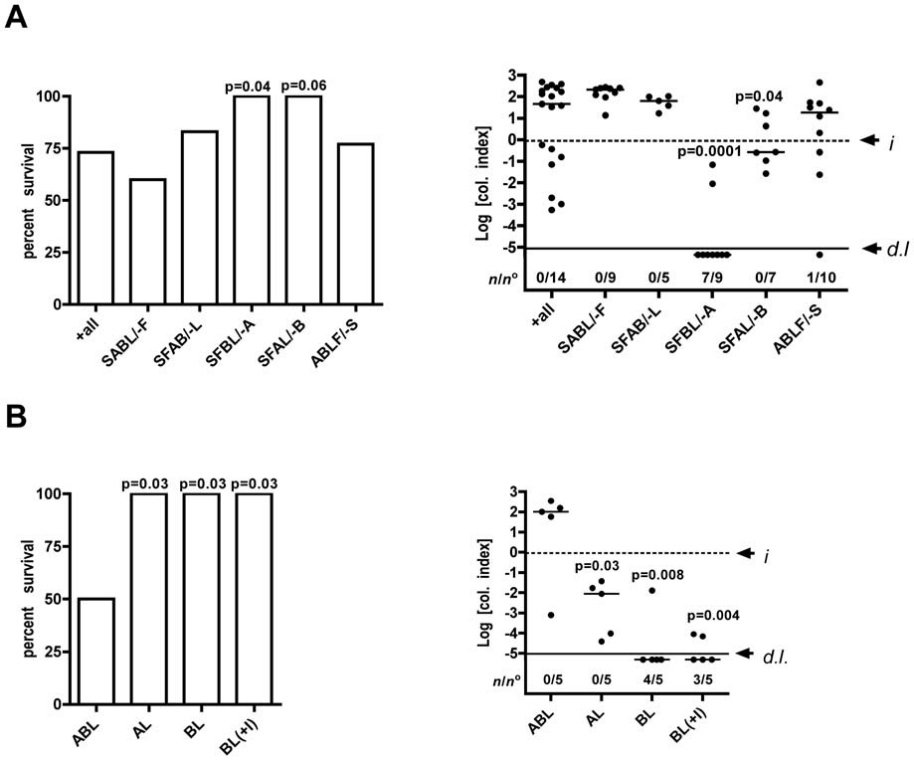
Inoculation strategy affects successful colonization of C57BL/6 mice. (A) Prior to inoculation, mice were treated as indicated on the X axis (S-1 mg/ml streptomycin in water; F-restriction of food overnight; A-anesthesia with ketamine-xylazine intraperitoneally at time of inoculation; B-intragastric delivery of sodium bicarbonate, and L-inoculation of bacteria in the logarithmic phase of growth. Control mice (+all) were infected using all five parameters. Mice were then inoculated with 1−5×10^7^ CFU *V. cholerae* El Tor O1 strain P27459 and colonization of the small intestine assessed after 20 hr. Data are pooled from 3 experiments so the input variation between experiments was normalized by dividing the recovered CFU by the input CFU and data are thereby plotted as a log colonization index with dashed line at 0 indicating that CFU recovered was identical to the input CFU (*i*). Markers above the dashed line are indicative of growth in the small intestine while markers below indicate clearance. Values below the solid line (*d.l.*) were below the detection limit of 500 CFU in the small intestine. The number of mice that cleared *V. cholerae* from the small intestine (*n*) over the total of mice in the group (*n*°) is also indicated. The experiment in panel B was performed as above except an additional parameter of anesthesia with inhaled isoflurane (+I) was added and data are pooled from two experiments. Significant and borderline P values are shown compared to control for survival (calculated by a χ2 test) and for colonization (calculated by a Mann-Whitney non-parametric t-test comparing medians).

Comparison of both survival rates and colonization 20 hr p.i. showed that food restriction (SABL/-F), growth phase of bacteria (SFAB/-L), and streptomycin pretreatment did not significantly affect successful colonization or survival. Survival rates in these groups were similar ranging from 60–83%, consistent with a dose of 1−5×10^7^ as near the LD_50_ and median colonization indices representing 10–200 fold growth (Fig. 1A). Although not quantitatively different than use of an overnight culture of *V. cholerae*, log phase growth was routinely used as the bacteria could be quantified more accurately by spectrometry to standardize inocula between different groups and experiments.

By contrast, in the non-anesthetized group (SFBL/-A), all the mice survived to 20 hr and colonization was poor with only 2 of 9 mice colonized above the detection limit (Fig. 1A). In the group that did not receive sodium bicarbonate (SFAL/-B), all the mice also survived and colonization was significantly lower than in the control group (p = 0.04) (Fig. 1A). These data indicated that anesthesia at the time of inoculation was the most significant factor promoting colonization and sodium bicarbonate also had a positive influence on colonization.

The previous experiment demonstrated parameters that were necessary for efficient colonization. To determine if anesthesia and bicarbonate treatment are sufficient to achieve colonization, one reference group was anesthetized with ketamine-xylazine and inoculated with sodium bicarbonate followed by log-phase bacteria (ABL). Two additional groups were either only anesthetized (AL/-B) or only treated with sodium bicarbonate (BL/-A). A third group was anesthetized with isoflurane in place of ketamine-xylazine (BL(+I)). The group that was both anesthetized with ketamine-xylazine and treated with sodium bicarbonate (ABL) had 50% lethality and the surviving mice were colonized at levels similar to mice treated with all inoculation parameters (Fig. 1B). Mice inoculated with use of only ketamine-xylazine or sodium bicarbonate showed significantly reduced colonization leading to increased survival (Fig. 1B). These data demonstrate that anesthesia with ketamine-xylazine and sodium bicarbonate are both necessary and sufficient to achieve high levels of early colonization of the small intestine. Surprisingly, anesthesia with isoflurane could not substitute for ketamine-xylazine (Fig. 1B). This result indicates that the positive effect of ketamine-xylazine on colonization is not simply associated with sedation, but rather is likely due to side effects of the drugs.

### A broad range of mouse strains can be used for studies of intestinal colonization

**Figure 2:**
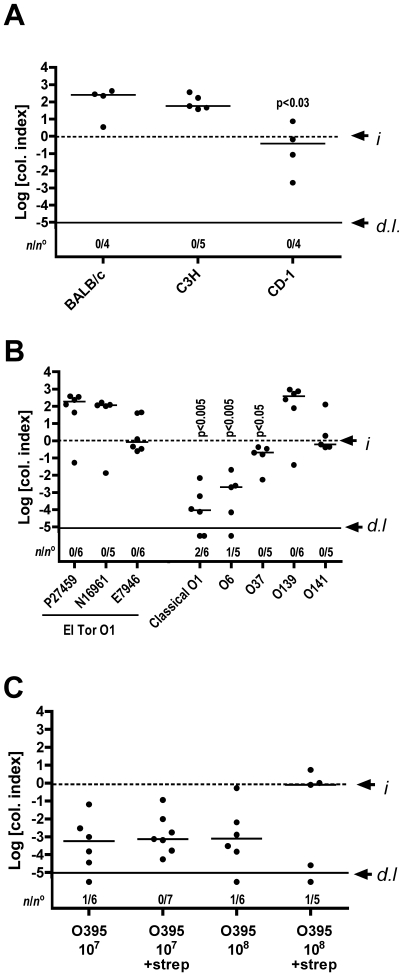
Effect of mouse and *V. cholerae* strain variation on colonization. (A) Mouse strains as indicated were inoculated with 1.1×10^7^ CFU El Tor O1 strain P27459. (B) C57BL/6 mice were inoculated with 0.5−1×10^7^ CFU of *V. cholerae* strain as indicated. (C) Streptomycin treated and non-treated C57BL/6 mice were inoculated with either 2−3×10^7^ or 1.5−3.9×10^8^ CFU of *V. cholerae* Classical O395. All mice were inoculated with the ABL strategy as described in the text and mice were assessed for colonization of the small intestine 20 hr after inoculation. Values are reported as a Col. Index as described in Fig. legend 1. The number of mice that cleared *V. cholerae* from the small intestine (*n*) over the total of mice in the group (*n*°) is also indicated. Only statistically significant p values calculated by a Mann-Whitney non-parametric t-test comparing medians are shown. P values in panels A and B are compared to El Tor O1 P27459 infection in C57BL/6 mice (Panel B) and in panel C to O395 control at 10^7^ CFU.

To determine whether intestinal infection with *V. cholerae* can be studied in mice other than C57BL/6 mice, two inbred mouse strains, BALB/c and C3H, and the outbred mouse strain CD-1 were inoculated using the ABL protocol with ∼10^7^ CFU El Tor O1 strain P27459. All 3 strains of mice were colonized, indicating that colonization is not restricted to one specific mouse strain (Fig. 2A). BALB/c and C3H mice showed colonization indices 2 log units above the inoculum similar to observed colonization of P27459 in C57BL/6 mice (Fig. 1B and 2B). By contrast bacterial numbers recovered from CD-1 mice were significantly lower than those from C57BL/6, BALB/c, and C3H mice (p = 0.03). Thus, the outbred CD-1 mice may have a reduced genetic susceptibility to *V. cholerae*. However, it should be noted that, at the time of inoculation, the age matched CD-1 mice had significantly higher body weights (25±0.9 g, p<0.001) compared to BALB/c and C3H mice (16.7±0.7 g and 18.5±1.2 g, respectively). Thus, it is possible the higher body weight of the CD-1 mice accounts for the observed differences in colonization levels.

### Both El Tor and some non-O1 *V. cholerae* strains can also colonize the small intestine

As previous studies were limited to El Tor bacteria, it was essential to determine if *V. cholerae* O1 of the Classical biotype or from serogroups other than O1 can colonize the small intestine of mice as well. C57BL/6 mice were inoculated using the ABL protocol with ∼10^7^ CFU of O1 and non-O1 *V. cholerae* strains and bacterial recovery was determined after 20 hr. As expected, El Tor O1 strain P27459 colonized at high levels about 2 log units above the inoculum. Other El Tor strains N16961 and E7946 also colonized. By contrast, bacterial numbers recovered from mice inoculated with Classical strain O395 were significantly lower (p< = 0.001) suggesting a severe defect in colonization (Fig. 2B).

Among non-O1 strains, the O139 strain MO10 was recovered at levels similar to P27459 (Fig. 2B), the O141 strain similar to E7946, and the O37 strain significantly lower at ∼10-fold below the inoculum (p = 0.03). However, bacterial numbers from mice inoculated with O6 were dramatically lower than P27459-inoculated mice (p = 0.002), colonizing with only low recovery of bacteria similar to the Classical strain.

The reduced colonization in mice infected with the Classical strain was pursued further. The infecting dose was increased and both streptomycin-treated and non-treated mice were compared (Fig. 2C). Although still below colonization levels normally observed with El Tor strains and not significantly different from controls, some mice inoculated with O395 (3/5) were colonized at levels near the inoculum in streptomycin treated mice given a higher dose. Thus, it is possible that any strain of *V. cholerae* can be used in mouse inoculation studies, but the infective dose both in the presence and absence of streptomycin must be first determined empirically.

### Colonization of mice inoculated with revised ABL protocol is still dependent upon accessory toxins

**Figure 3:**
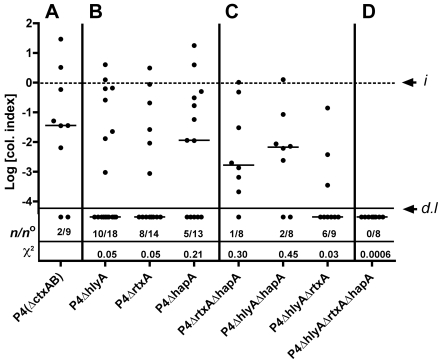
Colonization of the small intestine depends on accessory toxins. Data represent merged results from 4 experiments, wherein C57BL/6 mice were inoculated by the ABL strategy with a low dose between 4×10^5^ to 4×10^6^ CFU and colonization of the small intestine was assessed after 48 hr. Values are reported as a Col. Index as described in Fig. legend 1. Despite variation in input, there was no correlation between increased inoculum and improved colonization in these data sets. The number of mice that cleared the infection over the total mice in the experimental group is shown in the graph as number cleared (*n*) over number inoculated *n*°. Number of mice varies since single mutant knockout infections were repeated more often with more mice per group to assure that observed differences in clearance frequency initially observed were reproducible and not representative of experiment to experiment variation or due to differences in the inoculum.

In contrast to virulence and short-term colonization, experiments of long-term colonization require low dose inoculum so that the mice survive beyond 24 hr. Previously we showed that El Tor O1 strain P27459 inoculated at low dose (10^5^–10^6^ CFU) could persist in the small intestine at 2–3 log units below the inoculum dose up to 72 hr without being cleared [5]. This ability to establish persisting colonization did not depend on CT or toxin co-regulated pili (TCP), but did depend on accessory toxins [5]. Consistent with these findings, 78% of mice inoculated by the ABL protocol with CT deletion strain P4 remained colonized to 48 hr after low dose infection, but none of the mice inoculated with multitoxin deletion strain KFV101 (P4Δ*hlyA*Δ*rtxA*Δ*hapA*) remained colonized (Fig. 3A and 3D, Mann-Whitney, p<0.001, χ^2^ = 0.0006)). Thus, our revised inoculation protocol did not change the requirement for accessory toxin genes to establish a colonization that persists to 48 hr.

### Colonization of mice depends on hemolysin and MARTX

To define which of the three individual accessory toxin(s) caused the colonization defect of the multitoxin mutant strain KFV101, colonization of the small intestine by P4 mutants deleted of a single accessory toxin gene using the ABL inoculation strategy was performed (Fig. 3B). Merged data from four experiments showed that, contrary to consistent data wherein P4 colonizes and KFV101 (P4Δ*hlyA*Δ*rtxA*Δ*hapA*) does not colonize, the deletion of any single accessory toxin gene — *hlyA*, *rtxA*, or *hapA* — from P4 reduced the overall colonizing frequency to 8/18 (44%, χ^2^, p = 0.0502), 6/14 (43%, χ^2^, p = 0.0496), or 8/13 (62%, χ^2^, p = 0.211) mice, respectively, with the contributions of *hlyA* and *rtxA* borderline for statistical significance. When the single knockout strains did colonize, CFU recovered from the small intestine of colonized mice was not significantly different from P4 (Mann-Whitney, p = 0.26 to 0.48). Given results with single mutants, it was not surprising that a mutant deleted of both *hlyA* and *rtxA* was significantly defective for colonization (Mann-Whitney, p = 0.005; χ^2^, p = 0.030) further identifying *hlyA* and *rtxA* as the accessory toxins that promote colonization (Fig. 3C). By contrast, deletion of *hapA* from either the P4Δ*rtxA* or P4Δ*hlyA* strain did not further reduce colonization. Indeed, the loss of HA/protease slightly increased colonization, although the data are not significantly different from isogenic single mutants (Mann-Whitney, p = 0.18 to 0.40). These data indicated that none of the accessory toxin genes are absolutely essential for colonization, although hemolysin and MARTX significantly increase the number of mice successfully colonized. In the P4 strain that expresses all three toxins, the combined contribution of hemolysin and MARTX is sufficient to overcome a minor antagonistic effect from HA/protease.

## Discussion

At the outset of this study, we sought to define both the physical and genetic parameters that facilitate colonization of mice with *V. cholerae* so that the model can be used both for basic science investigation and vaccine development. One factor leading to successful colonization of adult mice was the administration of sodium bicarbonate to neutralize stomach acid. Surprisingly, the most prominent factor was the use of ketamine-xylazine as the anesthetic during inoculation. Ketamine and xylazine have many pharmacological side effects, including an influence on intestinal motility in large animals [9], [10]. However, studies in rats have shown that intestinal motility is not affected by ketamine [11]. The positive effect of ketamine-xylazine in mice may therefore be related to additional side effects of the drugs. It has been shown that ketamine alone, or in combination with xylazine, inhibits the host response to LPS leading to development of shock [12], [13]. Specifically, ketamine attenuates LPS-induced TNFα expression in the gastric mucosa and serum and decreases iNOS production throughout the intestine, particular the small intestine. TNFα in serum and the gastric mucosa return to basal level within 3 hr after injection of LPS [12], [13]. TNFα and iNOS are linked to innate immune responses including release of antimicrobial peptides and ROS. In the context of a bacterial infection, we propose that use of ketamine during inoculation facilitates initial colonization of the small intestine by inhibiting killing of the bacteria shortly after intragastric inoculation. In fact, a study performed in 1975 demonstrated that *V. cholerae* administered to mice orally was killed in the small intestine within 2 hr after inoculation [14]. Thus, ketamine may induce a transient immunodeficiency for about 3 hr, that in the case of *V. cholerae*, is absolutely essential for mice to be susceptible to small intestine colonization. Without ketamine, >10^9^ CFU *V. cholerae* and prior treatment with streptomycin were required to colonize mice; but even then, the bacteria predominantly localized to the large bowel [15], rather than the small intestine as seen in ketamine-treated mice [5].

The genetics of the bacterial and mouse strains also dramatically affected successful colonization. Among wild-type strains tested, El Tor O1 and O139 successfully colonized to 20 hr, suggesting these strains with pandemic potential are most suited for these types of studies although some non-O1/non-O139 strains colonized as well. By contrast, a Classical O1 strain poorly colonized and likely will require a high dose and streptomycin treatment for successful infection. It is known that Classical strains do not express a fully functional hemolysin [16], MARTX [17] or HA/protease [18]. Similarly, PCR analysis revealed the poorly colonizing O6 strain did not have an *hlyA* gene (data not shown). Thus, the absence of accessory toxins may account for reduced colonization by these strains. With regard to genetics of the mice, the outbred mouse strain CD-1 was less susceptible to colonization, however, it is possible this reflected the size of the mice rather than genetics. In any case, it seems as if any combination of bacterial strain and mouse strain might be used for these types of studies, but the effective dose of the test strain in age-matched mice will need to be routinely established.

Interestingly, among the accessory toxins, we found MARTX and hemolysin in combination account for successful colonization of El Tor O1 strains. Surprisingly, hemolysin and MARTX affected the number of mice colonized, but not the level of bacteria recovered from the small intestine of colonized mice. Thus, the purpose of the accessory toxins could be to actively defend against innate immune cells keeping the immune system suppressed during early infection allowing time for the bacteria to replicate and colonize. These toxins might function by directly targeting phagocytic cells. Hemolysin has been shown to affect neutrophils, while MARTX is known to cause actin crosslinking in macrophages [19], [20]. Alternatively, these toxins could function by targeting resident mast cells, macrophages and/or dendritic cells blocking cytokine and chemokine release reducing the influx of phagocytic cells that could aid in clearance of the bacteria.

In humans, it is predicted that the colonizing bacteria would next progress to synthesis of the primary virulence factors CT and TCP, resulting in the onset of cholera disease, generally 1–5 days after exposure to the bacterium [21]. However, in most instances, the bacteria would simply persist in the intestinal tract until cleared 5–10 days later by the acquired immune system, accounting for the large number of asymptomatic carriers after *V. cholerae* exposure [22].

Overall, our findings likely explain why other researchers have not been able to successfully colonize adult mice with *V. cholerae*. With the help of this defined model, one can now explore many new hypotheses in *V. cholerae*-host interaction, such as studying different genetic backgrounds and employing the various immunological tools that are available for mice. In particular, this mouse model will provide a means to test vaccine efficacy prior to human clinical trials of oral live attenuated vaccines. A recent report used oral challenge of vaccinated mice, but the challenge required a high inoculom in streptomycin and isoflurane-treated mice followed by monitoring CFU in stool for up to 17 days [15]. By contrast, using ketamine-treated mice, we have demonstrated that oral introduction of a live attenuated strain of *V. cholerae* induces protection from a lethal dose of *V. cholerae* [6], a much more convincing monitor of successful mucosal immunity.

## References

[1] Cash RA, Music SI, Libonati JP, Snyder MJ, Wenzel RP (1974). Response of man to infection with *Vibrio cholerae*. I. Clinical, serologic, and bacteriologic responses to a known inoculum.. J Infect Dis.

[2] Chiang SL, Mekalanos JJ (1999). *rfb* mutations in *Vibrio cholerae* do not affect surface production of toxin-coregulated pili but still inhibit intestinal colonization.. Infect Immun.

[3] Hsiao A, Liu Z, Joelsson A, Zhu J (2006). *Vibrio cholerae* virulence regulator-coordinated evasion of host immunity.. Proc Natl Acad Sci USA.

[4] Kirn TJ, Lafferty MJ, Sandoe CM, Taylor RK (2000). Delineation of pilin domains required for bacterial association into microcolonies and intestinal colonization by *Vibrio cholerae*.. Mol Microbiol.

[5] Olivier V, Salzman NH, Satchell KJ (2007). Prolonged colonization of mice by *Vibrio cholerae* El Tor O1 depends on accessory toxins.. Infect Immun.

[6] Olivier V, Haines GK,, Tan Y, Satchell KJ (2007). Hemolysin and the multifunctional autoprocessing RTX toxin are virulence factors during intestinal infection of mice with *Vibrio cholerae* El Tor O1 strains.. Infect Immun.

[7] Fullner KJ, Boucher JC, Hanes MA, Haines GK,, Meehan BM (2002). The contribution of accessory toxins of *Vibrio cholerae* O1 El Tor to the proinflammatory response in a murine pulmonary cholera model.. J Exp Med.

[8] Fullner KJ, Mekalanos JJ (1999). Genetic characterization of a new type IV pilus gene cluster found in both classical and El Tor biotypes of *Vibrio cholerae*.. Infect Immun.

[9] Freye E, Knufermann V (1994). [No inhibition of intestinal motility following ketamine-midazolam anesthesia. A comparison of anesthesia with enflurane and fentanyl/midazolam].. Anaesthesist.

[10] Herbert J (1998). Neurosteroids, brain damage, and mental illness.. Exp Gerontol.

[11] Suliburk JW, Gonzalez EA, Moore-Olufemi SD, Weisbrodt N, Moore FA (2005). Ketamine inhibits lipopolysacharide (LPS) induced gastric luminal fluid accumulation.. J Surg Res.

[12] Helmer KS, Cui Y, Chang L, Dewan A, Mercer DW (2003). Effects of ketamine/xylazine on expression of tumor necrosis factor-alpha, inducible nitric oxide synthase, and cyclo-oxygenase-2 in rat gastric mucosa during endotoxemia.. Shock.

[13] Helmer KS, Cui Y, Dewan A, Mercer DW (2003). Ketamine/xylazine attenuates LPS-induced iNOS expression in various rat tissues.. J Surg Res.

[14] Knop J, Rowley D (1975). Antibacterial mechanisms in the intestine. Elimination of *V. cholerae* from the gastrointestinal tract of adult mice.. Aust J Exp Biol Med Sci.

[15] Nygren E, Li BL, Holmgren J, Attridge SR (2009). Establishment of an adult mouse model for direct evaluation of the efficacy of vaccines against *Vibrio cholerae*.. Infect Immun.

[16] Alm RA, Stroeher UH, Manning PA (1988). Extracellular proteins of *Vibrio cholerae*: nucleotide sequence of the structural gene (*hlyA*) for the haemolysin of the haemolytic El Tor strain O17 and characterization of the *hlyA* mutation in the non-haemolytic classical strain 569B.. Mol Microbiol.

[17] Cordero CL, Sozhamannan S, Satchell KJ (2007). RTX toxin actin cross-linking activity in clinical and environmental isolates of *Vibrio cholerae*.. J Clin Microbiol.

[18] Hanne LF, Finkelstein RA (1982). Characterization and distribution of the hemagglutinins produced by *Vibrio cholerae*.. Infect Immun.

[19] Fullner KJ, Mekalanos JJ (2000). *In vivo* covalent crosslinking of actin by the RTX toxin of *Vibrio cholerae*.. EMBO J.

[20] Valeva A, Walev I, Weis S, Boukhallouk F, Wassenaar TM (2007). Pro-inflammatory feedback activation cycle evoked by attack of *Vibrio cholerae* cytolysin on human neutrophil granulocytes.. Med Microbiol Immunol.

[21] Sack DA, Sack RB, Nair GB, Siddique AK (2004). Cholera.. Lancet.

[22] Niyogi SG, Deb BC, Sircar BK, Sengupta PG, De SP (1979). Studies on cholera carriers and their role in transmission of the infection: a preliminary report.. Indian J Med Res.

[23] Mekalanos JJ (1983). Duplication and amplification of toxin genes in *Vibrio cholerae*.. Cell.

[24] Ghosh A, Saha DR, Hoque KM, Asakuna M, Yamasaki S (2006). Enterotoxigenicity of mature 45-kilodalton and processed 35-kilodalton forms of hemagglutinin protease purified from a cholera toxin gene-negative *Vibrio cholerae* non-O1, non-O139 strain.. Infect Immun.

[25] Li M, Shimada T, Morris JG, Sulakvelidze A, Sozhamannan S (2002). Evidence for the emergence of non-O1 and non-O139 *Vibrio cholerae* strains with pathogenic potential by exchange of O-antigen biosynthesis regions.. Infect Immun.

[26] Waldor MK, Mekalanos JJ (1994). ToxR regulates virulence gene expression in non-O1 strains of *Vibrio cholerae* that cause epidemic cholera.. Infect Immun.

[27] Bina J, Zhu J, Dziejman M, Faruque S, Calderwood S (2003). ToxR regulon of *Vibrio cholerae* and its expression in vibrios shed by cholera patients.. Proc Natl Acad Sci USA.

[28] Goldberg I, Mekalanos JJ (1986). Cloning of the *Vibrio cholerae recA* gene and construction of a *Vibrio cholerae recA* mutant.. J Bacteriol.

